# The Effect of Surgeon and Hospital Volume on Morbidity and Mortality After Femoral Shaft Fractures

**DOI:** 10.5435/JAAOSGlobal-D-22-00242

**Published:** 2023-05-03

**Authors:** Edward J. Testa, Peter G. Brodeur, Christopher J. Lama, Davis A. Hartnett, David Painter, Joseph A. Gil, Aristides I. Cruz

**Affiliations:** From the Department of Orthopaedic Surgery, Brown University, Warren Alpert School of Medicine, Providence, RI.

## Abstract

The aim of this study was to characterize the case volume dependence of both facilities and surgeons on morbidity and mortality after femoral shaft fracture (FSF) fixation.

**Methods:**

Adults who had an open or closed FSF between 2011 and 2015 were identified in the New York Statewide Planning and Research Cooperative System database. Claims were identified by International Classification of Disease-9, Clinical Modification diagnostic codes for a closed or open FSF and International Classification of Disease-9, Clinical Modification procedure codes for FSF fixation. Readmission, in-hospital mortality, and other adverse events were compared across surgeon and facility volumes using multivariable Cox proportional hazards regression, controlling for patient demographic and clinical factors. Surgeon and facility volumes were compared between the lowest and highest 20% to represent low-volume and high-volume surgeons/facilities.

**Results:**

Of 4,613 FSF patients identified, 2,824 patients were treated at a high or low-volume facility or by a high or low-volume surgeon. Most of the examined complications including readmission and in-hospital mortality showed no statistically significant differences. Low-volume facilities had a higher 1-month rate of pneumonia. Low-volume surgeons had a lower 3-month rate of pulmonary embolism.

**Conclusion:**

There is minimal difference in outcomes in relation to facility or surgeon case volume for FSF fixation. As a staple of orthopaedic trauma care, FSF fixation is a procedure that may not require specialized orthopaedic traumatologists at high-volume facilities.

Femoral shaft fractures (FSFs) are among the most common injuries that orthopaedic surgeons are required to manage.^1,2^ The femur is both the strongest tubular bone in the body and the principal load-bearing bone in the lower extremity, and even minor degrees of malunion or malalignment after a traumatic fracture can lead to limping, post-traumatic arthritis, and prolonged disability.^3,4^ Femoral diaphyseal fractures are often the result of high-energy forces and may be associated with multiple system injuries.^4^ With the high likelihood of orthopaedic surgeons encountering such fractures and the considerable risk of morbidity and mortality, optimization of FSF care and triage management is of utmost importance.

Prior trauma literature has shown that both hospital and surgeon surgical volumes can affect the clinical outcomes of certain orthopaedic procedures.^5-9^ In isolated proximal femur fractures, management by low-volume hospitals and surgeons have been associated with prolonged length of stay and higher rates of in-hospital mortality.^6,7^ Some studies investigating total joint arthroplasty have demonstrated that high-volume facilities and surgeons yield better outcomes and further defined that surgeons who conducted more than 35 cases had lower rates of early revision and postoperative dislocation.^10^ By contrast, some studies examining other orthopaedic procedures have identified improved outcomes by low-volume hospitals or no volume-outcome relationship at all with various orthopaedic procedures.^5,7,11,12^ Metcalfe et al^5^ found no notable difference in clinical outcomes for hip fractures treated at low-volume vs. high-volume centers, concluding that there is no patient safety imperative to limit hip fracture care to high-volume hospitals. The current body of literature demonstrates that some orthopaedic procedures exhibit volume-outcome relationships, whereas others may not, indicating that there may be a procedure-specific dependency.

To our knowledge, there have been no studies investigating the volume-outcome relationship for the surgical treatment of FSFs. These fractures are commonly encountered by orthopaedic surgeons of all practice models and, depending on the hospital site or surgeon availability, may be managed by a specialized traumatologist or by the surgeon on call, regardless of specialty training. Despite the potential disparities in outcomes this creates, few epidemiological or outcomes-based studies have been published on FSFs.^4^ The purpose of this study was to characterize the volume dependence of both hospitals and surgeons on the morbidity and mortality after the surgical treatment of FSFs. We hypothesize that patients receiving care at high-volume institutions and by high-volume surgeons will have lower postoperative complication and mortality rates than those of low-volume institutions and surgeons.

## Methods

Patients 18 years and older were identified in the New York Statewide Planning and Research Cooperative System (SPARCS) database from 2011 to 2015. SPARCS is an all-payer database collecting all inpatient and outpatient (emergency department, ambulatory surgery, and hospital-based clinic visits) claims in New York. This includes all International Classification of Diseases (ICD) diagnosis codes and ICD/Current Procedural Terminology procedure codes associated with all visits. Inpatient claims were first identified using the ICD-9-Clinical Modification (CM) FSF diagnosis codes (821.01, 821.11). Claims were then filtered by ICD-9-CM procedure codes to isolate patients who went on to receive internal fixation surgery (ICD-9 CM: 79.35, 79.15, 78.55). Only a patient's first operation was considered eligible for follow-up. Nonresidents of New York were not included in our analysis. Given ICD-9 coding was discontinued after the third quarter of 2015, only the first 3 quarters of 2015 were used because these statistics are still likely to be indicative of the low-to-high–volume comparison.

A unique surgeon and facility identifier was used to calculate the total number of procedures per surgeon and facility per year. Based on the total volume per year, surgeons and facilities were subject to the lowest 20% of volume, middle 60% of volume, or highest 20% of volume. The boundaries for lowest and highest 20% deviated slightly by year but were selected to minimize the difference from the 20% volume mark.

Patients were followed up to a maximum of 1 year postoperatively in the inpatient and outpatient settings. The 1-month, 3-month, and 12-month risks of interest were readmission, urinary tract infection, acute renal failure, cardiorespiratory arrest, pneumonia, acute stroke, surgical site infection, deep vein thrombosis, acute respiratory failure, pulmonary embolism (PE), cellulitis, wound complications, in-facility mortality, and revision surgery (see Supplemental Table 1, http://links.lww.com/JG9/A279 for codes used). SPARCS claim dates are listed as the first day of the month in which the service occurred because of the SPARCS deidentification policy. Therefore, if a complication occurred within the same month as the primary procedure, the time to complication was defined as 0.5 months.^12^

Patient demographics were compared separately across facility volume and surgeon volume using chi square analysis. Student *t*-tests were used to compare sample means, and Mann-Whitney *U* tests were used when appropriate when continuous data were found to be not normally distributed.

Multivariable Cox proportional hazards regression was used for the analysis of risk likelihood across the volume groups. Each complication was modeled separately while controlling for patient age, sex, race, ethnicity, Charlson Comorbidity Index (CCI), primary insurance type, ICD Injury Severity Score (ISS), and social deprivation index (SDI). Other race excludes White, Asian, and African American, but does include multiracial. The regression models assess the risk difference across surgeon and facility groups simultaneously by controlling for both in the same model. Two separate multivariable logistic regression models were used to assess the odds of receiving treatment by a high-volume surgeon or at a high-volume facility. The independent variables used were the same as the medical complication analysis. A multivariable linear regression model using the same variables as the medical complication analysis was used to assess the risk factors associated with an increased length of stay (LOS).

The CCI was calculated using the method described by Deyo et al.^13^ CCI was dichotomized to a score of 0 versus a score of ≥1. SDI, as described by Butler et al, was linked to each patient based on ZIP code. SDI provides a robust measure of social determinants of health not typically captured by healthcare administrative databases by converting the following categories to an index from 1 to 100: percent living in poverty, percent with less than 12 years of education, percent of single-parent household, percent living in a rented housing unit, percent living in an overcrowded housing unit, percent of households without a car, and percent of unemployed adults younger than 65 years. A higher SDI score equates to increased social deprivation. SDI data in this study were based on 2015 statistics.^14^ Survival ratios were calculated for each trauma ICD-9-CM code using the methods described by Osler et al^15^ across the entire data set before application to this model. Based on a patient's trauma diagnosis codes, the survival ratios associated with each code were then multiplied out to derive the ISS. Therefore, it should be noted that lower ISS scores equate to worse injuries as multiplying smaller survival ratios results in a lower ISS value.

A *P* value of <0.01 was considered significant across all statistical analyses. All analyses were conducted using SAS 9.4 (SAS).^12^

## Results

Of 4,613 FSF patients identified, 2,824 patients were treated at a high or low-volume facility or by a high or low-volume surgeon. The yearly facility volume ranged from 1 to 83 (mean: 9, median: 4) procedures. The yearly surgeon volume ranged from 1 to 27 (mean: 2, median: 1) procedures. The total number of procedures per year remained relatively constant with a range of 1,139 to 1,288 (894 through 3 quarters of 2015). The range for the number of procedures used as the boundary for the lowest 20% of volume by facility was 6 to 18 (5 through 3 quarters of 2015) and for the highest 20% was 51 to 62 (48 through 3 quarters of 2015). Low-volume facilities accounted for 992 procedures, and high-volume facilities accounted for 1,022 procedures. The number of low, middle, and high-volume facilities was 128, 36, and 3, respectively. The range of the number of procedures used as the boundary for the lowest 20% of volume by surgeon was 2 for all years from 2011 to 2014 (1 through 3 quarters of 2015) and for the highest 20% was 9 to 12 (8 through 3 quarters of 2015). Low-volume surgeons accounted for 929 procedures, and high-volume surgeons accounted for 975 procedures (Tables 1 and 2). The number of low, middle, and high-volume surgeons was 692, 373, and 11, respectively.

**Table 1:** Patient Demographics and Characteristics, by Facility Volume

Factor	Low Volume (n = 992)	High Volume (n = 1,022)	*P* Value
Age, mean (SD)	77 (71.9, 19.3)	41 (46, 23.4)	**<0.0001**
Sex, n (%)			
Female	727 (73.3)	400 (39.1)	**<0.0001**
Male	265 (26.7)	622 (60.9)	—
Ethnicity, n (%)			
Non-Hispanic	898 (90.5)	1004 (98.2)	**<0.0001**
Hispanic	94 (9.5)	18 (1.8)	—
Race, n (%)			
White	782 (78.8)	804 (78.7)	0.9295
Asian	28 (2.8)	9 (0.9)	**0.0012**
African American	80 (8.1)	149 (14.6)	**<0.0001**
Other	102 (10.3)	60 (5.9)	**0.0003**
Primary insurance, n (%)			
Private	241 (24.3)	668 (65.4)	**<0.0001**
Federal	714 (72)	274 (26.8)	**<0.0001**
Self-pay	18 (1.8)	41 (4)	**0.0035**
Charlson score, n (%)			
0	421 (42.4)	683 (66.8)	**<0.0001**
≥1	571 (57.6)	339 (33.2)	—
ISS, median (mean, SD)	0.96 (0.95, 0.05)	0.94 (0.87, 0.15)	**<0.0001**
SDI, median (mean, SD)	49 (48.7, 30)	42 (46.6, 29.3)	0.1548

ISS = Injury Severity Score, SDI = social deprivation index. Bold represents statistically significant data.

**Table 2:** Patient Demographics and Characteristics, by Surgeon Volume

Factor	Low Volume (n = 929)	High Volume (n = 975)	*P* Value
Age, mean (SD)	67 (61.7, 24.3)	48 (49.7, 24.5)	**<0.0001**
Sex, n (%)			
Female	567 (61)	431 (44.2)	**<0.0001**
Male	362 (39)	544 (55.8)	—
Ethnicity, n (%)			
Non-Hispanic	833 (89.7)	902 (92.5)	0.029
Hispanic	96 (10.3)	73 (7.5)	—
Race, n (%)			
White	660 (71)	676 (69.3)	0.4147
Asian	26 (2.8)	28 (2.9)	0.9235
African American	118 (12.7)	154 (15.8)	0.0539
Other	125 (13.5)	117 (12)	0.3406
Primary insurance, n (%)			
Private	370 (39.8)	560 (57.4)	**<0.0001**
Federal	501 (53.9)	314 (32.2)	**<0.0001**
Self-pay	28 (3)	56 (5.7)	**0.0037**
Charlson score, n (%)			
0	473 (50.9)	645 (66.2)	**<0.0001**
≥1	456 (49.1)	330 (33.9)	—
ISS, median (mean, SD)	0.95 (0.9, 0.14)	0.95 (0.9, 0.11)	0.0124
SDI, median (mean, SD)	50 (50.9, 31.4)	52 (52.2, 31.7)	0.3065

ISS = Injury Severity Score, SDI = social deprivation index. Bold represents statistically significant data.

Several demographic differences were noted to be statistically significant. Low-volume facilities and surgeons had age distributed toward older ages relative to high-volume facilities and surgeons (Tables 1 and 2). Low-volume facilities had an increased incidence of female sex, Hispanic ethnicity, Asian race, federal insurance, having ≥1 Charlson comorbidity, and being distributed toward less injury severity (Table 1). Low-volume surgeons had increased incidence of female sex, federal insurance, and having ≥1 Charlson comorbidity (Table 2).

Several postoperative complications were analyzed at 1, 3, and 12 months. Most of the examined complications including readmission and in-hospital mortality showed no statistically significant differences between low or high-volume surgeons or facilities (Table 4). Low-volume facilities had a higher 1-month rate of pneumonia (Table 3). Low-volume surgeons had a lower 3-month rate of PE (Table 4).

**Table 3:** Risk of Complication After Femur Fracture Fixation, by Facility Volume

Factor	Low Volume (n = 992)	High Volume (n = 1,022)	Hazard Ratio (99% CI)	*P* Value
Readmission				
1 month	139 (14)	89 (8.7)	1.265 (0.822-1.948)	0.16
3 month	196 (19.8)	139 (13.6)	1.139 (0.8-1.622)	0.3439
12 month	328 (33.1)	245 (24)	1.128 (0.859-1.48)	0.2552
Urinary tract infection				
1 month	224 (22.6)	97 (9.5)	1.268 (0.851-1.888)	0.1249
3 month	240 (24.2)	111 (10.9)	1.2 (0.824-1.749)	0.2115
12 month	283 (28.5)	131 (12.8)	1.15 (0.813-1.624)	0.2992
Acute renal failure				
1 month	89 (9)	48 (4.7)	0.982 (0.552-1.746)	0.9354
3 month	96 (9.7)	53 (5.2)	0.921 (0.531-1.6)	0.7025
12 month	135 (13.6)	67 (6.6)	0.998 (0.69-1.444)	0.9925
Cardiorespiratory arrest				
1 month	10 (1)	8 (0.8)	0.997 (0.613-1.621)	0.9859
3 month	14 (1.4)	8 (0.8)	1.602 (0.337-7.622)	0.4363
12 month	22 (2.2)	9 (0.9)	1.895 (0.504-7.131)	0.2141
Pneumonia				
1 month	58 (5.9)	41 (4)	1.961 (1.018-3.775)	**0.0081**
3 month	73 (7.4)	53 (5.2)	1.619 (0.9-2.91)	0.0344
12 month	101 (10.2)	68 (6.7)	1.415 (0.85-2.355)	0.079
Acute stroke				
1 month	22 (2.2)	14 (1.4)	1.178 (0.47-2.956)	0.6463
3 month	24 (2.4)	17 (1.7)	1.409 (0.469-4.229)	0.4216
12 month	36 (3.6)	24 (2.4)	1.189 (0.591-2.391)	0.6267
Surgical site infection				
1 month	18 (1.8)	27 (2.6)	0.924 (0.357-2.393)	0.8315
3 month	22 (2.2)	32 (3.1)	0.947 (0.396-2.263)	0.8719
12 month	24 (2.4)	36 (3.5)	1.028 (0.454-2.324)	0.9317
Deep vein thrombosis				
1 month	43 (4.3)	38 (3.7)	1.622 (0.783-3.358)	0.0872
3 month	53 (5.3)	43 (4.2)	1.812 (0.927-3.542)	0.0224
12 month	62 (6.3)	49 (4.8)	1.671 (0.892-3.131)	0.0352
Acute respiratory failure				
1 month	32 (3.2)	66 (6.5)	1.205 (0.602-2.411)	0.4879
3 month	36 (3.6)	70 (6.9)	1.163 (0.597-2.264)	0.5592
12 month	45 (4.5)	74 (7.2)	1.208 (0.648-2.253)	0.4346
Pulmonary embolism				
1 month	23 (2.3)	26 (2.5)	1.637 (0.662-4.045)	0.1608
3 month	25 (2.5)	27 (2.6)	1.768 (0.735-4.251)	0.0944
12 month	29 (2.9)	28 (2.7)	1.85 (0.798-4.287)	0.0595
Cellulitis				
1 month	24 (2.4)	21 (2.1)	1.082 (0.41-2.851)	0.8349
3 month	29 (2.9)	27 (2.6)	1.03 (0.427-2.482)	0.9315
12 month	40 (4)	41 (4)	0.874 (0.421-1.813)	0.6345
Wound comp				
1 month	23 (2.3)	26 (2.5)	0.981 (0.392-2.454)	0.9559
3 month	25 (2.5)	29 (2.8)	1.017 (0.429-2.41)	0.9606
12 month	35 (3.5)	35 (3.4)	1.11 (0.52-2.369)	0.7235
In-hospital mortality				
1 month	35 (3.5)	14 (1.4)	1.525 (0.539-4.311)	0.2958
3 month	49 (4.9)	16 (1.6)	1.702 (0.666-4.347)	0.1444
12 month	73 (7.4)	23 (2.3)	1.669 (0.764-3.644)	0.0911
Revision				
1 month	11 (1.1)	14 (1.4)	1.199 (0.306-4.69)	0.7319
3 month	18 (1.8)	19 (1.9)	1.013 (0.35-2.935)	0.9748
12 month	40 (4)	43 (4.2)	0.794 (0.392-1.608)	0.3994

CI = confidence interval. Bold represents statistically significant data.

Hazard ratios are adjusted for physician volume, age, sex, race, ethnicity, primary insurance type, Charlson Comorbidity Index, and social deprivation index.

**Table 4:** Risk of Complication After Femur Fracture Fixation, by Surgeon Volume

Factor	Low Volume (n = 929)	High Volume (n = 975)	Hazard Ratio (99% CI)	*P* Value
Readmission				
1 month	105 (11.3)	87 (8.9)	0.919 (0.6-1.409)	0.612
3 month	168 (18.1)	133 (13.6)	1.002 (0.71-1.413)	0.9903
12 month	278 (29.9)	246 (25.2)	0.901 (0.694-1.171)	0.3062
Urinary tract infection				
1 month	177 (19.1)	109 (11.2)	1.024 (0.703-1.491)	0.8696
3 month	191 (20.6)	121 (12.4)	1.027 (0.718-1.47)	0.8458
12 month	231 (24.9)	140 (14.4)	1.093 (0.785-1.521)	0.4884
Acute renal failure				
1 month	82 (8.8)	39 (4)	1.4 (0.782-2.506)	0.1371
3 month	87 (9.4)	43 (4.4)	1.396 (0.797-2.444)	0.1256
12 month	108 (11.6)	57 (5.9)	1.296 (0.888-1.891)	0.1791
Cardiorespiratory arrest				
1 month	8 (0.9)	4 (0.4)	1.297 (0.789-2.131)	0.1782
3 month	9 (1)	4 (0.4)	1.314 (0.222-7.78)	0.6924
12 month	13 (1.4)	4 (0.4)	1.88 (0.363-9.735)	0.3225
Pneumonia				
1 month	65 (7)	43 (4.4)	0.874 (0.493-1.552)	0.5469
3 month	76 (8.2)	54 (5.5)	0.859 (0.508-1.454)	0.4571
12 month	91 (9.8)	69 (7.1)	0.84 (0.523-1.349)	0.3424
Acute stroke				
1 month	10 (1.1)	14 (1.4)	0.368 (0.102-1.322)	0.0441
3 month	12 (1.3)	18 (1.9)	0.355 (0.113-1.121)	0.0203
12 month	22 (2.4)	25 (2.6)	0.536 (0.212-1.351)	0.0822
Surgical site infection				
1 month	28 (3)	22 (2.3)	1.151 (0.503-2.633)	0.6611
3 month	33 (3.6)	25 (2.6)	1.236 (0.572-2.669)	0.4787
12 month	42 (4.5)	35 (3.6)	1.084 (0.557-2.109)	0.7558
Deep vein thrombosis				
1 month	48 (5.2)	33 (3.4)	1.131 (0.583-2.196)	0.6328
3 month	52 (5.6)	41 (4.2)	0.935 (0.506-1.727)	0.7768
12 month	58 (6.2)	48 (4.9)	0.886 (0.497-1.58)	0.5902
Acute respiratory failure				
1 month	50 (5.4)	45 (4.6)	0.848 (0.469-1.536)	0.4752
3 month	51 (5.5)	46 (4.7)	0.851 (0.473-1.533)	0.4803
12 month	56 (6)	49 (5)	0.858 (0.485-1.515)	0.4869
Pulmonary embolism				
1 month	19 (2.1)	32 (3.3)	0.488 (0.212-1.122)	0.0264
3 month	19 (2.1)	34 (3.5)	0.438 (0.193-0.996)	**0.0096**
12 month	22 (2.4)	35 (3.6)	0.473 (0.216-1.035)	0.0138
Cellulitis				
1 month	27 (2.9)	21 (2.2)	1.056 (0.439-2.539)	0.8724
3 month	30 (3.2)	25 (2.6)	1.049 (0.462-2.383)	0.8797
12 month	43 (4.6)	37 (3.8)	1.091 (0.551-2.159)	0.7435
Wound comp				
1 month	23 (2.5)	23 (2.4)	0.814 (0.344-1.929)	0.5398
3 month	30 (3.2)	24 (2.5)	1.02 (0.461-2.255)	0.9486
12 month	42 (4.5)	28 (2.9)	1.238 (0.61-2.511)	0.4377
In-hospital mortality				
1 month	31 (3.3)	12 (1.2)	1.272 (0.456-3.545)	0.5462
3 month	40 (4.3)	15 (1.5)	1.279 (0.512-3.193)	0.4881
12 month	51 (5.5)	23 (2.4)	1.099 (0.51-2.369)	0.752
Revision				
1 month	8 (0.9)	12 (1.2)	0.716 (0.18-2.84)	0.5321
3 month	13 (1.4)	17 (1.7)	0.809 (0.274-2.393)	0.6151
12 month	36 (3.9)	34 (3.5)	1.285 (0.633-2.608)	0.3621

CI = confidence interval. Bold represents statistically significant data.

Hazard ratios are adjusted for physician volume, age, sex, race, ethnicity, primary insurance type, Charlson Comorbidity Index, and social deprivation index.

**Figure 1:**
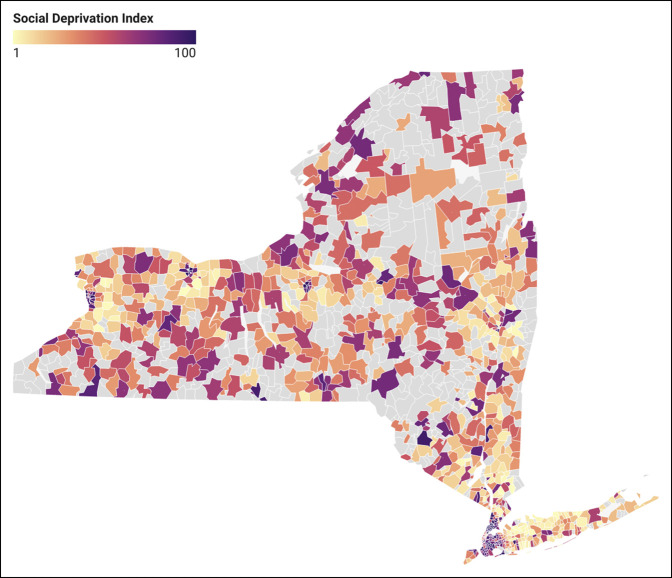
Map showing social deprivation index scores by ZIP code in New York (1-100). Higher social deprivation index scores represent higher social deprivation. ZIP codes in gray had no femur fractures included in the study.

**Figure 2:**
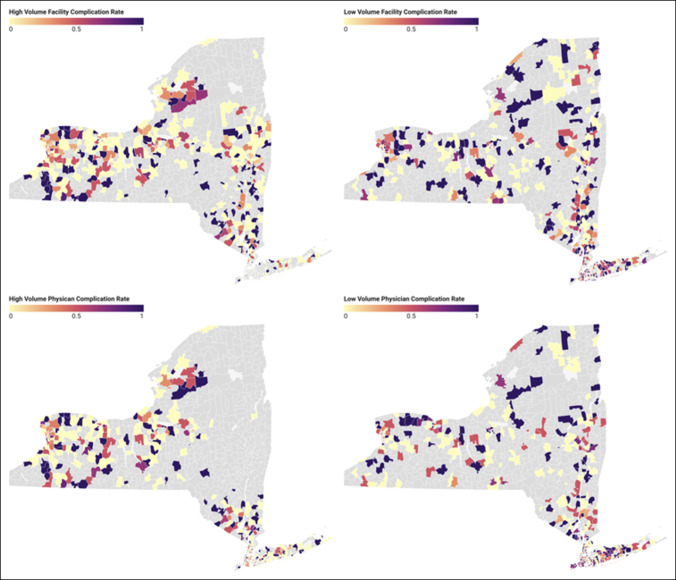
Maps showing 3-month complication rates by facility and surgeon volumes by ZIP codes. Gray ZIP codes had either no complications or no femur fracture cases included in this study.

**Figure 3:**
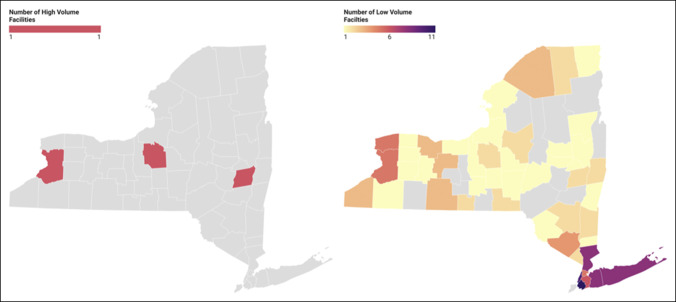
Maps showing the count of high and low-volume facilities by county in New York. Gray ZIP codes did not have a high or low-volume facility that managed a femur shaft fracture.

Figure 1 illustrates how the SDI varies across New York ZIP codes, with darker areas representing higher social deprivation. Figure 2 illustrates the rate of 3-month complications among patients by ZIP code stratified by facility and surgeon volumes. While also having higher SDI scores, Western Long Island had increased density of patients who endured complications and were treated by a low-volume surgeon or at a low-volume facility (Figures 1 and 2). Figure 3 shows the number of high and low-volume facilities for FSF across New York. When compared with Figure 1, high-volume facilities are located in areas with less social deprivation (Figures 1 and 3).

The logistic regression analysis assessing the odds of receiving treatment by a high-volume surgeon showed decreased odds for those treated at a low or middle-volume facility. A higher ISS (less severe injuries) had increased odds of being treated by a high-volume surgeon (Supplemental Table 1, http://links.lww.com/JG9/A280).

The logistic regression analysis assessing the odds for receiving treatment at a high-volume facility showed decreased odds for older ages and female sex. Asian, African American, and other races had decreased odds of treatment at a high-volume facility compared with White race. Similarly, Hispanic ethnicity had decreased odds compared with non-Hispanic ethnicity. Federal insurance had decreased odds compared with private insurance. Those from areas with high social deprivation had decreased odds of being treated at a high-volume facility. Those treated by a low or middle-volume surgeon had decreased odds of treatment at a high-volume facility. Finally, keeping in mind that those with lower ISS have worse injuries, the logistic regression showed those with a higher ISS (or less severe injuries) had decreased odds of receiving care at a high-volume facility (Supplemental Table 2, http://links.lww.com/JG9/A280).

The multivariable linear regression for predicting length of stay showed that an increased length of stay was associated with older age, African American race, having one or more Charlson comorbidities, and worse injuries as specified by the negative coefficient for ISS. Female patients were associated with a decreased length of stay. Low/middle-volume facilities or surgeons did not affect length of stay when compared with high-volume facilities or surgeons, respectively.

## Discussion

This study used a large statewide database to investigate the relationship between both hospital and surgeon volumes on morbidity and mortality after surgical management of FSFs. Most of the complications analyzed were found not to be associated with either hospital or surgeon volume, with similar rates of readmission, surgical site infection, wound complications, revisions, and mortality between the observed groups. Lower volume facilities were observed to have a markedly higher 1-month rate of pneumonia in patients after surgical management of a FSF while higher volume surgeons saw a higher 3-month rate of PE. These findings are positive indicators that treatment location and operating surgeon do not largely affect surgical outcomes in the management of FSF.

The primary finding of this study was a general lack of notable differences in outcomes based on hospital and surgeon volumes, although certain notable differences, such as those relating to pulmonary complications, were observed. Pulmonary complications after femoral shaft and other long bone fractures are well-appreciated problems and can lead to notable morbidity and mortality.^16-18^ The incidence of pulmonary complications after either reamed or unreamed intramedullary nailing for FSFs ranges from roughly 2% to 14.6% in the literature, but can vary depending on several factors such as time to fixation and degree of trauma severity.^18-20^ In our comparison of femoral shaft fixation patients managed at high-volume versus low-volume facilities, markedly different complication rates were seen at 1 month for pneumonia, with higher rates at low-volume facilities (Table 3). Lower volume hospitals have previously been associated with higher rates of pneumonia after fracture repair and total hip arthroplasty (THA).^7,21^ Possibly, more comorbid patients in these hospitals when compared with high-volume facilities is the primary factor contributing to differential complication rates; this study identified a markedly greater number of comorbidities in the lower volume hospital population, suggesting that patient baseline health in these hospitals may be more important to postoperative outcomes than hospital volume (Table 1). Another possible explanation for the higher rates of pneumonia at low-volume facilities is that low-volume hospitals may have inferior access to certain aspects of high-level care, including specialty facilities, physiotherapy, and medical specialists.^21^ As a result, higher volume hospitals may have greater opportunities to identify and solve issues before the development of certain complications.^21-23^ Contrarily, low-volume surgeons were observed to have lower rates of PE at the 3-month postoperative point. Given that the prophylaxis for venous thromboembolism after femur fractures is largely standardized, it seems unlikely that surgeon volume is directly responsible for a disparity in PE rates. It is possible that individualized cases of particularly complicated polytraumas are being managed by higher volume surgeons or being transferred by lower volume surgeons, contributing to increased rates of PE among higher volume surgeons. Complication rates are otherwise largely comparable between surgeon groups.

No notable differences in mortality rates were observed between either high and low-volume hospitals or surgeons. Although high-volume surgeons saw lower rates of 12-month mortality from 2.4% to 5.5%, the difference between the cohorts was nonsignificant (*P* = 0.752). The relationship between patient volume and mortality after femoral fracture has been extensively studied in the context of hip fractures, although there has not been similar investigation into FSF fixation.^7,12,24-26^ Browne et al^7^ identified no difference in mortality based on hospital volume, but identified an increased risk of in-hospital mortality in patients who underwent hip fracture fixation by low-volume surgeons. Similar results were found by Forte et al,^24^ who examined Medicare claims for patients who experienced intertrochanteric hip fractures and found that while the very highest volume hospitals demonstrated superior in-hospital mortality, the results did not suggest that the outcomes at different locations were substantial enough to alter patient care. The authors did note that lower volume surgeons saw markedly higher mortality rates than the highest volume surgeons, further suggesting that practitioner volume is of greater importance than hospital volume when managing hip fractures. By contrast, a 2017 study by Okike et al^25^ examined over 14,000 patients in the Kaiser Permanente healthcare system who underwent hip fracture repair and found no association between either hospital or surgeon volume and patient mortality. These findings regarding surgeon volume were supported by a 2021 investigation of the SPARCS database by Testa et al^12^ specific to peritrochanteric hip fractures that found no notable difference in mortality between high and low-volume surgeons. Of note, they found an increase in mortality associated with low-volume hospitals, a unique finding attributed to possible differences in staff and hospital protocols at lower volume facilities. Studies into the effect of hospital and patient volume are generally inconsistent, with added variables of differing definitions of high volume and low volume. A 2018 systematic review found hospital volume to be a better predictor of hip fracture repair outcomes than surgeon volume, but noted a paucity of quality, standardized studies examining surgeon volume.^8^ Beyond hip fractures, a similar SPARCS study examining surgical fixation of typical diaphyseal tibial fractures found no association of outcomes with hospital or surgeon volume.^26^ The present study is the only investigation into the relationship between volume and outcomes of FSFs and identified no notable differences in mortality between hospital volume levels and surgeon volume levels. While there may exist some correlation of hospital or surgeon volume with patient mortality after the management of orthopaedic trauma, these do not seem to be profound enough to alter the direction of acute trauma patients toward different centers or surgeons.

Of note, this study observed secondary findings related to disparities in patient populations. Patients who were older, female, non-White, non-Hispanic, from lower socioeconomic areas, and on federal insurance were less likely to be treated at high-volume hospitals. However, these variables were not notable when assessing the factors that influence treatment by high-volume surgeons. Although these demographic variables were not observed to be associated with adverse outcomes after FSF fixation, this disparity in who is likely to be treated at high versus low-volume hospitals is highly relevant to other orthopaedic procedures. A retrospective review of the SPARCS database by Maceroli et al^27^ found that patients undergoing THA for femoral neck fractures saw improved mortality and decreased 90-day complication rates at the highest volume centers. Racial and socioeconomic disparities in outcomes after major fractures have been previously identified in the literature, including hip fractures and fragility fractures.^28,29^ Additional investigation into the relationship between patient demographics and negative postoperative outcomes in the context of facility volume may be warranted, given the notable association with lower volume hospitals, although this association was not observed after FSF fixation.

This study found that an increased length of stay was not markedly associated with either hospital or surgeon volume. This finding is not surprising given similar rates of complications between volume cohorts, especially when controlling for patient variables. However, LOS was found to be associated with older age, African American race, having one or more Charlson comorbidities, and worse injuries as specified by the negative coefficient for ISS. An increased LOS places an added burden on both the patient and the hospital system. A retrospective study by Pendleton et al^30^ examining length of stay after isolated FSF found increased time to surgery and need for placement after leaving the hospital as major contributors to LOS. The authors noted that in these patients, early medical/radiographic evaluation preoperatively and early evaluation of social factors postoperatively can be most valuable in reducing LOS. Although complication rates are similar in these patient populations, particular consideration in hospitals, regardless of facility volume, may help reduce length of stay after FSF.

In contrast to the present findings of traumatic fracture management, studies into the outcomes of elective orthopaedic procedures have repeatedly found inverse relationships between surgical volume and adverse outcomes. THA has demonstrated an association of higher hospital or surgeon volume with lower mortality rates in multiple studies, with many also demonstrating lower postoperative complication rates.^31-35^ Similar relationships have been observed in elective unicondylar and total knee arthroplasty^36-39^ as well as spinal deformity surgery in both adult and pediatric patients.^40-42^ These trends are not unexpected given the relative complexity of procedures such as arthroplasty or scoliosis correction, which require subspecialty training and extensive exposure to minimize complications. The elective nature of these procedures also serves to help standardize the patient population, with high-comorbidity or frail patients selected out. By contrast, common orthopaedic trauma cases are key components of residency and fellowship education, often with substantial exposure during training and the expectation that typical cases could be managed in practice without any subspecialty training experience. This was observed to be common practice in the present study because there were far more lower volume surgeons (692) compared with high-volume surgeons (11). Furthermore, given the difference in the number of low-volume and high-volume surgeons, a patient may be more likely to receive care by a low-volume surgeon at any point in time.

FSFs are predominantly noncomminuted simple AO type A fractures, often managed surgically with an antegrade intramedullary nail.^4^ These fractures are routinely managed by orthopaedic trainees during residency, with an Accreditation Council for Graduate Medical Education minimum of 25 surgical femoral or tibial shaft fractures required during residency.^43^ In addition, when treating a diverse population of trauma patients, variables such as comorbidities, associated polytrauma, severity of injury, and time to treatment may play much larger roles than hospital or surgeon volume.^25^ The results of this study support the expectation that typical orthopaedic trauma can be well-managed by orthopaedic surgeons at all varieties of hospitals, regardless of exposure volume.

This study has potential limitations inherent to the use of a retrospective administrative database. Accurate and comprehensive coding of patient comorbidities is not guaranteed, and while the potential for missed coding is mitigated by the incentive for hospitals to include all relevant diagnoses that could increase admission acuity and reimbursement, comorbidities deemed nonessential can go under-reported in trauma patients.^44^ We were unable to determine whether complications during the original hospital stay occurred before or after surgery, and, therefore, could result in increased reporting of postoperative complications. In addition, only medical complications were accessible for analysis, with disparities in orthopaedic outcomes such as radiographic and functional outcomes unable to be assessed in this study. The amount of low-volume facilities and low-volume surgeons was much greater than high-volume facilities and surgeons. The small number of high-volume facilities and surgeons makes it more difficult to assess whether differences in complications could be attributed to the volume-outcome relationship or could be attributed to the unique features among the few surgeons or facilities. Similarly, having a much larger number of low-volume facilities and surgeons introduces much more variability when attempting to assess the volume-outcome relationship. The SPARCS database is limited to patients within New York State, which limits broad extrapolation of conclusions to the United States or world populations, although the diversity of patients and providers present in a statewide database of the fourth most populous state allows for relative confidence in expanding results. We were unable to exclude patients in the database who were transferred from one hospital to another, potentially over-representing a higher risk population resulting in more data on complications and poor outcomes.

Despite these potential limitations, this study has important implications in the management of orthopaedic trauma patients. Taken as a whole, there are small differences in outcomes between both high and low-volume hospitals and surgeons, the clinical significance of which are up for debate. This suggests that FSFs may not need to be preferentially directed toward high-volume centers or high-volume surgeons for surgical management. As a staple of orthopaedic trauma care, femoral shaft fixation is a procedure that may not require specialized orthopaedic traumatologists at high-volume facilities, suggesting that urgent attention without overt concern for where the patient is directed is the optimal management for this traumatic injury.

## Supplementary Material
