# Supplementary material for: The Effect of Surgeon and Hospital Volume on Morbidity and Mortality After Femoral Shaft Fractures
Source: J Am Acad Orthop Surg Glob Res Rev. 2023 May 3;7(5):e22.00242. doi: 10.5435/JAAOSGlobal-D-22-00242 (PMC10162792; doi:10.5435/JAAOSGlobal-D-22-00242)
Supplement: Supplementary file 1 [file jagrr-7-e22.00242-s001.docx]

| **Supplemental Table 1.** Multivariable logistic regression for the odds of receiving treatment by a high volume surgeon | | |
| --- | --- | --- |
|  | **Odds Ratio**  **(99% CI)** | **P-value** |
| Age | 0.999 (0.993 - 1.006) | 0.8147 |
| Sex |  |  |
| Males | - | - |
| Females* | 0.994 (0.771 - 1.282) | 0.9516 |
| Race |  |  |
| White | - | - |
| Asianᵠ | 1.143 (0.631 - 2.073) | 0.5617 |
| African Americanᵠ | 1.014 (0.713 - 1.442) | 0.9189 |
| Otherᵠ | 1.158 (0.793 - 1.69) | 0.3192 |
| Ethnicity |  |  |
| Non-Hispanic Ethnicity | - | - |
| Hispanic Ethnicityᵞ | 1.177 (0.759 - 1.827) | 0.3386 |
| Primary Insurance |  |  |
| Private | - | - |
| Federalᵟ | 0.765 (0.574 - 1.019) | 0.0162 |
| Worker’s Compensationᵟ | 1.187 (0.691 - 2.039) | 0.415 |
| Self-Payᵟ | 1.626 (0.964 - 2.743) | 0.0167 |
| Charlson Score |  |  |
| CCI = 0 | - | - |
| CCI ≥ 1ᶲ | 0.87 (0.675 - 1.12) | 0.1545 |
| SDI | 1.002 (0.998 - 1.006) | 0.1552 |
| Fracture Type |  |  |
| Closed Fracture | - | - |
| Open Fracture^£^ | 1.027 (0.715 - 1.473) | 0.8517 |
| ISS | 6.056 (2.263 - 16.201) | **<.0001** |
| Facility Volume |  |  |
| Low (bottom 20%)^π^ | 0.007 (0.003 - 0.017) | **<.0001** |
| Middle (middle 60%)^π^ | 0.106 (0.083 - 0.136) | **<.0001** |
| High (highest 20%) | - | - |
| \| *compared to males \| \| --- \| \| ᵠcompared to white race \| \| ᵞcompared to non-Hispanic ethnicity \| \| ᵟcompared to private insurance \| \| ᶲcompared to CCI = 0  ^£^compared to closed fracture \| \| ^π^compared to high volume facilities \| | | |
