# Supplementary material for: The Effect of Surgeon and Hospital Volume on Morbidity and Mortality After Femoral Shaft Fractures
Source: J Am Acad Orthop Surg Glob Res Rev. 2023 May 3;7(5):e22.00242. doi: 10.5435/JAAOSGlobal-D-22-00242 (PMC10162792; doi:10.5435/JAAOSGlobal-D-22-00242)
Supplement: Supplementary file 2 [file jagrr-7-e22.00242-s002.docx]

| **Supplemental Table 2.** Multivariable logistic regression for the odds of receiving treatment at a high volume facility | | |
| --- | --- | --- |
|  | **Odds Ratio**  **(99% CI)** | **P-value** |
| Age | 0.979 (0.973 - 0.986) | **<.0001** |
| Sex |  |  |
| Males | - | - |
| Females* | 0.77 (0.594 - 0.997) | **0.0091** |
| Race |  |  |
| White | - | - |
| Asianᵠ | 0.149 (0.056 - 0.393) | **<.0001** |
| African Americanᵠ | 0.543 (0.375 - 0.788) | **<.0001** |
| Otherᵠ | 0.337 (0.213 - 0.533) | **<.0001** |
| Ethnicity |  |  |
| Non-Hispanic Ethnicity | - | - |
| Hispanic Ethnicityᵞ | 0.131 (0.064 - 0.267) | **<.0001** |
| Primary Insurance |  |  |
| Private | - | - |
| Federalᵟ | 0.594 (0.443 - 0.797) | **<.0001** |
| Worker’s Compensationᵟ | 0.642 (0.349 - 1.181) | 0.0611 |
| Self-Payᵟ | 0.554 (0.303 - 1.015) | 0.012 |
| Charlson Score |  |  |
| CCI = 0 | - | - |
| CCI ≥ 1ᶲ | 1.189 (0.912 - 1.551) | 0.093 |
| SDI | 0.995 (0.991 - 0.999) | **0.0024** |
| Fracture Type |  |  |
| Closed Fracture | - | - |
| Open Fracture^£^ | 1.263 (0.867 - 1.838) | 0.1096 |
| ISS | 0.121 (0.047 - 0.31) | **<.0001** |
| Surgeon Volume |  |  |
| Low (bottom 20%)^π^ | 0.063 (0.044 - 0.09) | **<.0001** |
| Middle (middle 60%)^π^ | 0.086 (0.067 - 0.112) | **<.0001** |
| High (highest 20%) | - | - |
| \| *compared to males \| \| --- \| \| ᵠcompared to white race \| \| ᵞcompared to non-Hispanic ethnicity \| \| ᵟcompared to private insurance  ᶲcompared to CCI = 0 \| \| ^£^compared to closed fracture  ^π^compared to high volume surgeons \| | | |
